# Development of a Fully Optimized Convolutional Neural Network for Astrocytoma Classification in MRI Using Explainable Artificial Intelligence

**DOI:** 10.3390/jimaging11100343

**Published:** 2025-10-02

**Authors:** Christos Ch. Andrianos, Spiros A. Kostopoulos, Ioannis K. Kalatzis, Dimitris Th. Glotsos, Pantelis A. Asvestas, Dionisis A. Cavouras, Emmanouil I. Athanasiadis

**Affiliations:** Medical Image and Signal Processing Laboratory, Department of Biomedical Engineering, University of West Attica, 12241 Athens, Greece; chandrianos@uniwa.gr (C.C.A.); skostopoulos@uniwa.gr (S.A.K.); ikalatzis@uniwa.gr (I.K.K.); dimglo@uniwa.gr (D.T.G.); pasv@uniwa.gr (P.A.A.); cavouras@uniwa.gr (D.A.C.)

**Keywords:** astrocytoma, convolutional neural network, hyperparameters optimization, explainable artificial intelligence

## Abstract

Astrocytoma is the most common type of brain glioma and is classified by the World Health Organization into four grades, providing prognostic insights and guiding treatment decisions. The accurate determination of astrocytoma grade is critical for patient management, especially in high-malignancy-grade cases. This study proposes a fully optimized Convolutional Neural Network (CNN) for the classification of astrocytoma MRI slices across the three malignant grades (G2–4). The training dataset consisted of 1284 pre-operative axial 2D MRI slices from T1-weighted contrast-enhanced and FLAIR sequences derived from 69 patients. To provide the best possible model performance, an extensive hyperparameter tuning was carried out through the Hyperband method, a variant of Successive Halving. Training was conducted using Repeated Hold-Out Validation across four randomized data splits, achieving a mean classification accuracy of 98.05%, low loss values, and an AUC of 0.997. Comparative evaluation against state-of-the-art pre-trained models using transfer learning demonstrated superior performance. For validation purposes, the proposed CNN trained on an altered version of the training set yielded 93.34% accuracy on unmodified slices, which confirms the model’s robustness and potential use for clinical deployment. Model interpretability was ensured through the application of two Explainable AI (XAI) techniques, SHAP and LIME, which highlighted the regions of the slices contributing to the decision-making process.

## 1. Introduction

Central Nervous System (CNS) tumors include a wide range of neoplasms, with gliomas representing the most common type of primary brain tumor, accounting for approximately 60% of all cases. Among gliomas, astrocytomas represent a major subtype, originating from astrocytes. The World Health Organization (WHO) classifies astrocytomas into four grades (G1–G4) based on histopathological characteristics and genetic alterations, including IDH mutation status, reflecting their level of malignancy and biological aggressiveness. Grades 1 and 2 are considered low-grade gliomas, with Grade 1 including pilocytic astrocytoma, typically regarded as benign. In contrast, Grades 3 and 4 are classified as high-grade gliomas, with Grade 4 encompassing glioblastoma IDH-wildtype, the most aggressive diffuse astrocytic tumor, as well as astrocytoma IDH-mutant, CNS WHO Grade 4. Accurate determination of astrocytoma grade is critical for patients’ therapy planning and prognosis estimation [1,2,3,4,5,6].

Recent studies have widely focused on the binary classification of gliomas into low-grade (LGG) and high-grade (HGG), as this distinction reflects tumor aggressiveness and influences treatment planning. Gilanie et al. [7] proceeded to design and tune a CNN in an empirical way to distinguish between LGG/HGG. Their experiment included both the BraTs-2017 public dataset and a locally developed dataset containing multi-sequence MRI images. Three different input formats were evaluated: the original image, skull-stripped images, and regions of interest (ROIs) for both datasets. The highest performance was achieved using the ROIs, with an accuracy of 98.52% on the local dataset and 96.40% on the benchmark dataset. Another representative approach involves the use of robust pre-trained CNN architectures. In the study [8], gliomas classification into LGG and HGG was addressed as one of two main tasks, alongside tumor segmentation. The researchers employed a fine-tuning strategy using BraTs-2020 dataset, incorporating T1-CE, T2, and FLAIR sequences from 369 patients. Their methodology involved fusing three MRI sequences into an additive Red–Green–Blue (RGB) color model to form composite input images. MobileNetV2 achieved the highest classification performance among the tested CNNs, reaching an accuracy of 99.85% on the fused dataset, approximately 6% higher than using only T2-weighted images, demonstrating the benefit of multi-sequence fusion.

Apart from the binary classification of gliomas into LGG and HGG, other studies focus on the more specific determination of the WHO glioma grade, addressing multi-classification tasks. For example, Rizwan et al. [9] developed a CNN-based method for brain tumor type and glioma grade classification. For the grading task, researchers utilized MRI data consisting of 516 images from 73 cases, in the T1-weighted sequence. A key step in the pre-processing phase was the application of a Gaussian filter, which was selected as the most suitable option, according to BRISQUE quality assessment metric. The empirically designed and tuned CNN achieved a classification accuracy of 97.14% at glioma grading. To improve generalization and avoid overfitting, data augmentation techniques were also applied. The empirically designed and tuned CNN achieved a classification accuracy of 97.14% in glioma grading. Additionally, a recent approach [10] employed three customized CNN architectures, each dedicated to a specific task: tumor detection, brain tumor classification, and glioma grading. For the grading task, a total of 4720 images from T1-weighted CE and FLAIR sequences were used. The hyperparameters of the networks were optimized using the Grid Search method. The proposed CNN for glioma grading achieved an accuracy of 98.56%, outperforming models trained using pre-trained architectures.

In a more specialized approach, some studies have focused on the astrocytomas classification into Low and High grade, aiming to provide a more accurate and targeted diagnosis of this specific type of glioma subtype. For example, Chen et al. [11] applied machine learning techniques using radiomic features to distinguish between patients with Low and High grade astrocytomas. The dataset was obtained from the Neurosurgery Department of West China Hospital and included T1-weighted CE MRI slices from 175 cases. Following segmentation by two specialists, 40 radiomic features were extracted using the LifeX software package (Version 6.20). Among the various combinations of feature selection methods and classifiers evaluated, the combination of LASSO for feature selection and LDA for classification yielded the best performance, with an accuracy of 72.90% and an Area Under the Curve (AUC) of 0.825.

A more fine-grained methodology involves the direct grading of astrocytoma images according to the WHO classification for CNS tumors. Gilanie et al. [12] proposed an automated method for astrocytoma grading using a customized CNN. For this multi-class classification task, MRI data were collected from 180 patients at the Department of Radiology BVHB, Pakistan, including T1-weighted, T2-weighted, and FLAIR sequences. The methodology involved tumor segmentation via the k-means clustering algorithm, guided by ground truth annotations. Data augmentations were applied by rotating selected slices that exhibited the largest tumor regions. The CNN was then trained to classify the regions of interest, achieving an overall accuracy of 96.56%, predicting all astrocytoma grades (G1–4).

This study introduces a fully optimized CNN framework tailored for the classification of tumorous astrocytoma grades (G2–G4). The innovation lies in the simultaneous optimization of both the network architecture and its hyperparameters using Hyperband, a state-of-the-art method for searching over wide parameter spaces. Unlike existing approaches, the proposed model operates with minimal or no pre-processing requirements. To further evaluate the robustness of the optimized architecture, data augmentation techniques were applied after the dataset was split, affecting only the training set. This approach allowed the system to be trained on an altered version of the slices, while testing was based on the original, unaltered data. Additionally, explainable AI (XAI) techniques, specifically SHAP and LIME, are employed to enhance model interpretability, providing transparent predictions through the analysis of both local and global explanations.

## 2. Materials and Methods

### 2.1. Data Collection

This study was conducted using astrocytoma 2D cross-sectional MRI scans obtained from two publicly available image collections hosted by The Cancer Imaging Archive (TCIA). The first collection, the Repository of Molecular Brain Neoplasia Data (REMBRANDT), consisted of 110,020 MRI slices from 130 patients diagnosed with brain neoplasms, including low and high-grade astrocytomas [13]. The second collection, The Cancer Genome Atlas Low Grade Glioma (TCGA-LGG) was part of the broader TCGA initiative and contained a total of 241,183 MRI and CT slices from 199 patients diagnosed with low-grade gliomas [14]. These collections were selected following an extensive search in publicly accessible medical imaging repositories. The primary inclusion criteria were the presence of tumor annotations following the WHO classification of central nervous system (CNS) tumors and the clear identification of the tumor within the slices, ensuring that the neoplasms were visually distinguishable.

Despite the large number of available image data across the two collections, only a limited subset met the specific requirements of this study. Among all glioma cases, a substantial proportion was diagnosed as oligodendroglioma, a category that was excluded from the study’s scope, which focused on astrocytomas. Furthermore, many patient slices contained incomplete data or artifacts resulting from motion or other quality issues, which led to their exclusion for use in the present study.

Following this selection process, the final dataset was formed by 1284 axial pre-treatment 2D cross-sectional MRI scans, acquired from Gadolinium T1-weighted contrast-enhanced (T1 CE) and FLAIR sequences. The dataset included 456 Grade 2, 408 Grade 3, and 420 Grade 4 astrocytoma slices, obtained from 28, 22, and 19 patients, respectively. Grade 4 astrocytoma cases correspond to glioblastomas in the REMBRANDT annotations; however, in the present study, they are reported as Grade 4 astrocytomas, as the dataset lacks information regarding IDH mutation status. The adoption of this terminology was driven by the necessity to ensure consistency with the histopathological grading framework, which was already available in the dataset.

**Figure 1:**
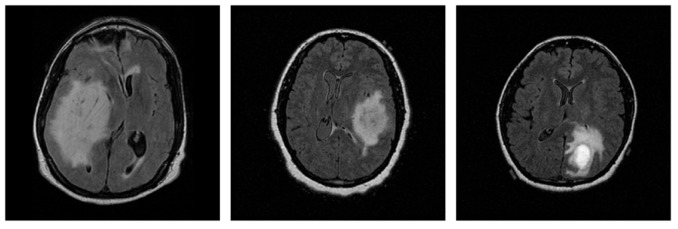
Representative 2D MRI slices of astrocytomas from each grade in the dataset: Grade 2 (**left**), Grade 3 (**center**), and Grade 4 (**right**).

The selected data originate from various MRI scanners manufactured by Siemens and GE, with slice thickness ranging from 2.5 to 5.0 mm, thereby providing variability and removing scanner-related bias limitation in the proposed method. A representative sample of each grade is presented in Figure 1.

**Figure 2:**
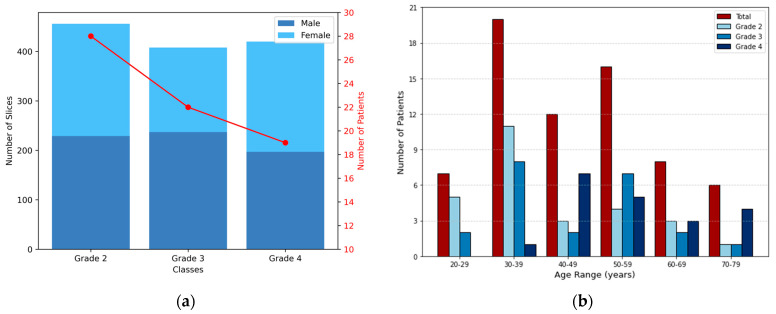
Distribution of 2D MRI slices and patient demographics across astrocytoma grades. (**a**) Bars represent the number of slices per class, corresponding to the left y-axis, while also illustrating the gender distribution. The red line indicates the number of patients in each grade, corresponding to the right y-axis. (**b**) Each group of bars corresponds to an age interval (10 years range), with the total number of patients shown in dark red and the grade-specific distributions shown in shades of blue.

The distribution of MRI slices and patient demographics across the different astrocytoma grades is presented in Figure 2. In Figure 2a, color-coded bars indicate the number of slices per class (left y-axis), showing the gender distribution for visualization purposes. The red line represents the number of patients per grade (right y-axis). Figure 2b illustrates the age distribution in 10-year intervals. Dark red bars represent the total number of patients per interval, while shades of blue indicate grade-specific counts. The dataset consisted exclusively of adult patients (20–79 years), thus avoiding variability related to growth and development in pediatric cases.

### 2.2. Data Pre-Processing

The pre-processing stage focused on normalizing the intensity values of the image arrays and standardizing the image dimensions to ensure compatibility with deep learning models. This stage was kept minimal because CNNs are capable of directly processing raw images, extracting relevant features with minimal intervention, thus reducing the need for extensive pre-processing. The final slice size was set to 224 × 224 pixels, a commonly used resolution in pre-trained CNN models. To achieve this, a two-step resizing procedure was implemented.

Regarding intensity normalization, all 2D cross-sectional MRI scans were scaled to the range [0, 255]. This approach allows the model to focus on biologically relevant regions, preventing the impact of variations in scanning or imaging parameters. According to the literature, this step is particularly important when working with datasets collected from multiple institutions [15].

**Figure 3:**
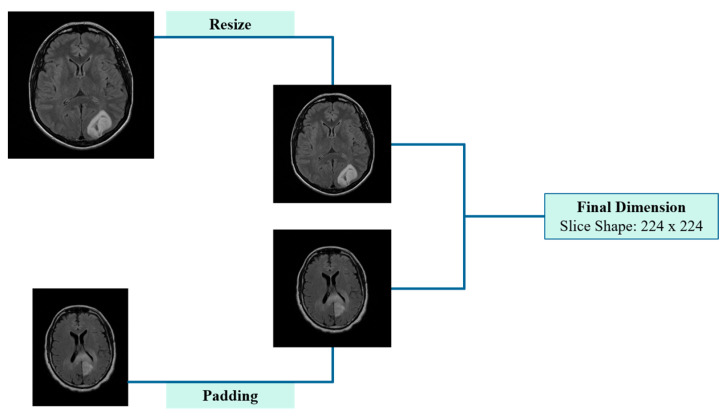
Schematic representation of pre-processing applied to 2D cross-sectional MRI scans, including padding and resizing.

Initially, slices with one smaller dimension (e.g., 208 × 256 pixels) underwent zero-padding, where black (zero-intensity) pixels were symmetrically added to the smaller dimension, resulting in a square format. This step ensured aspect ratio preservation and prevented geometric distortion. Notably, this pre-processing approach is widely used in preparing input data for CNN, as it preserves classification accuracy while simultaneously reducing the training time [16]. The subsequent step involved resizing all slices, including those originally sized 256 × 256 or 512 × 512 pixels, to the target size, ensuring consistency in spatial resolution while maintaining anatomical integrity. Zero-padding and resizing procedures are visually summarized in Figure 3, after which the data were fully prepared for input into the CNN. The entire pre-processing workflow is visually summarized in Figure 3, after which the data were fully prepared for input into the CNN.

### 2.3. Convolutional Neural Network—Hyperparameter Optimization

In this study, a CNN was developed for the classification of MRI slices into the three astrocytoma grades. In radiology, CNNs provide significant advantages over traditional image analysis techniques by leveraging hierarchical feature learning to automatically extract spatial and structural patterns [17]. The proposed CNN architecture consists of convolutional and pooling layers for progressive feature extraction, followed by fully connected layers for the final classification. The architecture of the CNN, which includes the sequence and configuration of its layers, along with the selection of initial hyperparameters, plays a critical role in determining the network’s classification performance.

According to the literature, CNN hyperparameters can be broadly categorized as architectural, regularization, and training. Architectural hyperparameters define the structure of the CNN, determining the configuration of the network. These include parameters such as the number of filters, kernel sizes, the number of dense layers, and the nodes that include [18]. All these parameters can affect the way that the network performs the feature extraction and the details that are taken into account in analyzing and creating the image representations.

Regularization parameters are designed to reduce the risk of bias and overfitting, thereby ensuring that the model is characterized by good generalization. A widely used regularization technique is the dropout, which is applied after the feature extraction layers and before the fully connected (dense) layers to prevent co-adaptation of neurons [19].

Lastly, training hyperparameters, also referred to as fine-adjustment parameters, govern the optimization process during the model’s training phase and have a significant impact on the overall performance. These hyperparameters include the choice of optimizer, the learning rate and the batch size, all of which determine the speed and the effectiveness of the training process [20,21].

To efficiently explore a wide range of hyperparameter configurations and identify the most effective CNN model architecture, this study employed the Hyperband optimization algorithm, which is particularly suitable for large-scale hyperparameter search problems. Initially, Hyperband generates random hyperparameter configurations based on the available computational resources, organizing them into distinct groups called brackets. Within each bracket, the Successive Halving algorithm is applied, which iteratively evaluates the performance of each configuration and eliminates the underperforming ones, progressively allocating more resources to the most promising combinations. This strategy effectively integrates random search with early stopping, achieving a balance between the exploration of the search space and the exploitation of the best-performing configurations [22].

### 2.4. Image Augmentation Methods

Data augmentation techniques have been widely adopted in deep learning workflows within the medical domain, as they enhance model generalization and help prevent overfitting, particularly in cases of limited training data. By artificially increasing the diversity of training data through label-preserving transformations, data augmentation reduces the model’s reliance on the original dataset, thereby enhancing its robustness. Commonly employed augmentation methods include geometric transformations, intensity modifications, and elastic deformations [23,24].

**Figure 4:**
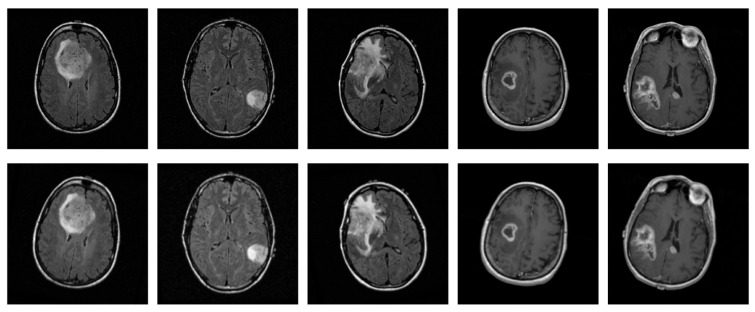
Representative original MRI slices from the training set (**top row**) and their corresponding augmented versions (**bottom row**), generated using random rotation, flipping, and shifting.

In medical imaging, including MRI scans, various sources of variation, such as artifacts caused by patient movement or acquisition inconsistencies, can affect image quality and, consequently, diagnostic assessments. These factors underscore the importance of evaluating the robustness of the proposed CNN under realistic perturbations, in addition to its generalization capabilities. For this purpose, mild geometry-based augmentations were applied exclusively to the training set, with each original slice replaced by a single augmented version, thereby ensuring that no augmented slice appeared in the test set, while simulating potential variability in real-world data. Specifically, random translations were performed by shifting the image up to 0.25% of its width or height, while random zoom operations were performed within a ±1% range. Additionally, random rotations were introduced, in 5% of a full cycle, corresponding to angular shifts of ±18 degrees. These augmentation parameters were configured within a safe and widely adopted range, as suggested in the literature [25,26], ensuring the preservation of diagnostic information, while evaluating the model’s performance under spatial variability. Figure 4 displays five original sample slices alongside their augmented counterparts, generated using random rotation, flipping, and shifting.

### 2.5. Explainable AI Methods

Interpretability is a critical requirement in the healthcare domain, especially when machine learning models are involved in decision-making processes. In recognition of this, the European Union introduced the Artificial Intelligence Act (AAI), which emphasizes the necessity of explainability, either through intrinsic interpretability or post hoc explanation methods [27]. Intrinsic or ad hoc interpretability refers to models that are inherently understandable and follow a transparent decision-making framework, typically including traditional machine learning algorithms such as decision trees or linear models. In contrast, post hoc explanation methods are associated with complex, non-transparent models that function as “black boxes”, including CNNs [28].

Considering the importance of interpretability in clinical decision support, two post hoc algorithms were employed to gain insights into the predictions of the proposed CNN model. The first model, SHAP (SHapley Additive exPlanations), is based on cooperative game theory and employs Shapley values to attribute the model’s prediction to individual input features. This technique provides a theoretical, model-agnostic approach, quantifying the contribution of each feature to the final prediction, thereby offering a deeper understanding of the model’s decision-making process [29]. In this study, SHAP was applied using Deep Explainer to analyze representative samples from each of the three astrocytoma grades involved in the classification task. By highlighting positively and negatively contributive pixels, this method enabled the identification of specific slice regions that influenced the proposed CNN’s predictions.

The second method employed was LIME (Local Interpretable Model-Agnostic Explanations), a technique capable of analyzing the predictions of any machine learning model. LIME generates local perturbations of the input data by creating slight variations of the original sample. For image data, the image is first segmented into distinct ‘superpixels’, which are homogeneous regions characterized by similar pixel properties. Perturbations are then applied by randomly turning these superpixels on or off, effectively altering parts of the image. Each perturbed instance is then weighted using a Gaussian (RBF) kernel, which assigns higher importance to samples more similar to the original input. This weighting ensures that the explanation focuses on the local behavior of the model [30]. In the context of this study, LIME was applied to explain the predictions of the proposed CNN for classifying astrocytoma grades, allowing the identification of specific 2D cross-sectional slice regions that contribute positively or negatively to each predicted class.

An important step following the implementation of XAI techniques was the assessment of reproducibility and agreement between SHAP and LIME subsequent to multiple trainings of the CNN. The evaluation focused on the tumor regions, where the similarity between the outputs of the two algorithms was quantified, thereby providing overlap measures with important clinical value. Since SHAP produces continuous values and LIME binary segmentations, the top 15% of pixels with the highest SHAP values were selected to enable comparison with the superpixels generated by LIME. Similarity then was assessed using widely adopted metrics in medical image analysis, including Jaccard Index, Dice Coefficient, Pearson Correlation, and Cosine Similarity, providing a quantitative evaluation [31]. These metrics capture similarity from various perspectives, combining spatial overlap with correlation-based measures.

### 2.6. Experimental Setup and Evaluation Strategy

The proposed CNN training was achieved using an NVIDIA TESLA P100 GPU with 16 GB RAM. For the astrocytoma grading classification task, the dataset was subjected to Repeated Hold-Out Validation, also referred as Random Subsampling, a special case of Monte Carlo Cross-Validation in which the train/test split ratio remains fixed [32]. Specifically, the dataset was four times randomly divided into 75% for training and 25% for testing/validation. For each random division, the system utilized a training using three different seeds, resulting in a total of 12 independent runs. This strategy aimed at reliable training while keeping the randomness factor absent, reducing bias and examining the reproducibility of training results. Although validation techniques vary in the literature, the utilization of Repeated Hold-Out Validation was motivated by the low computational complexity and robustness on variability datasets, while also allowing meaningful comparison with other studies [33,34].

To evaluate the system’s performance during training, accuracy and loss values were recorded across all runs. The results are reported as mean validation accuracy, accompanied by the corresponding sample-based standard deviation and the 95% confidence interval (CI), providing insight into performance consistency.

## 3. Results

### 3.1. Hyperparameter Optimization

Among all experimental trials, the highest classification accuracy of 98.75% was recorded on the test set, designating the selected architecture as the optimal model. The finalized network architecture comprised three sequential blocks, each consisting of double convolutional layers followed by a max-pooling layer. This structure was relatively simple, computationally efficient, and demonstrated superior performance for the specific classification task.

The optimal architectural hyperparameters of the proposed CNN model are summarized in Table 1. Moreover, Table 2 presents the optimal dropout rate identified via the Hyperband algorithm, which served as the primary regularization parameter explored in this study. For the optimization process, the Adam optimizer was employed, selected for its adaptive capabilities and widespread use in deep learning applications, offering an effective balance between computational efficiency and convergence speed. The training hyperparameters examined, along with their optimal values, are detailed in Table 3.

### 3.2. Optimized CNN Training Results

**Figure 5:**
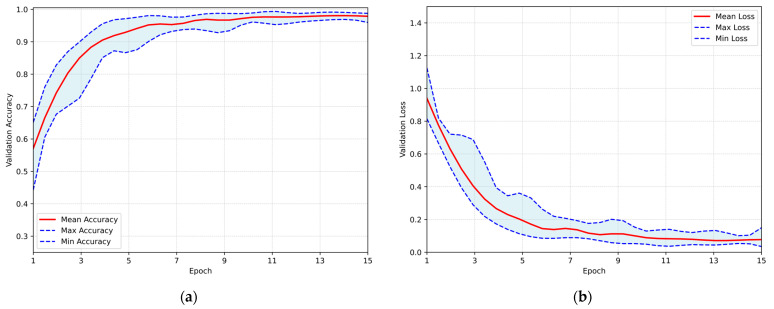
Overall performance of the proposed CNN evaluated using the Repeated Hold-Out strategy: (**a**) validation accuracy per training epoch; (**b**) validation loss per training epoch.

After completing the hyperparameter optimization process, the proposed CNN was trained utilizing the Repeated Hold-Out Validation method, as previously described. Each training session consisted of 15 epochs, with 465 iterations per run, resulting in a total of 5580 iterations across all runs. The overall system performance is illustrated in Figure 5, which presents the validation accuracy and loss over the epochs. In both subfigures, the red line represents the mean performance, and the shaded blue area corresponds to the performance range. The maximum average accuracy of 98.05% was achieved at the 14th epoch, with a standard deviation of ±0.72% and a corresponding 95% CI ranging from 97.60% to 98.51%. Concurrently, the minimum loss of 0.071 was occurred at the 13th training epoch, thereby demonstrating exceptional performance at the astrocytoma grades classification task.

**Figure 6:**
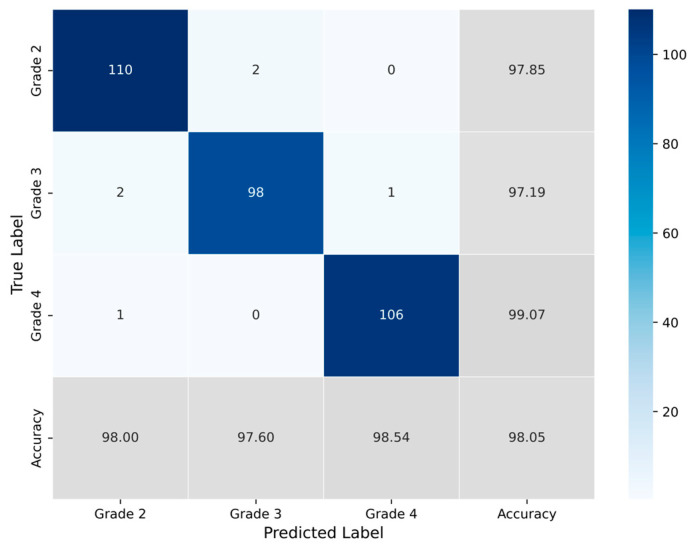
Average confusion matrix obtained across all runs of the Proposed CNN model, illustrating the classification performance for the three astrocytoma grades.

**Figure 7:**
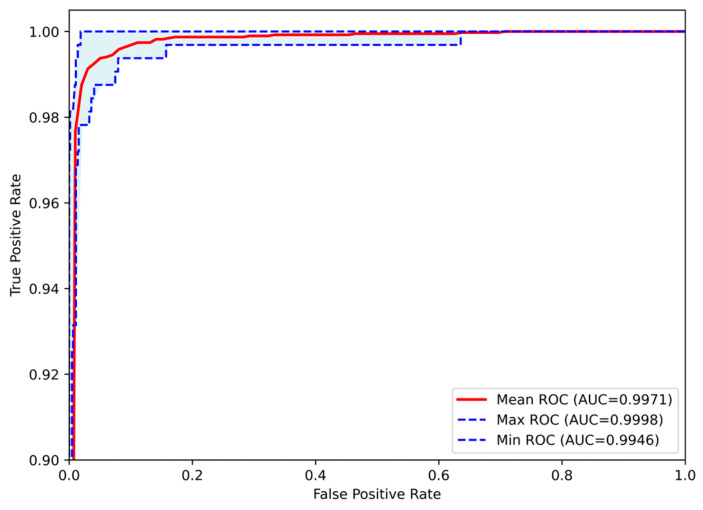
Receiver Operating Characteristic (ROC) curves of the Proposed CNN model for astrocytoma grade classification, aggregated across all runs. The mean ROC (AUC = 0.9971) reflects the model’s overall discriminative performance, while the maximum (AUC = 0.9998) and minimum (AUC = 0.9946) curves indicate the range of variability across runs.

The classification process was followed by the application of evaluation metrics on the validation set, to assess the system’s performance and robustness. The mean confusion matrix is presented in Figure 6, providing detailed insights into the classification accuracy for each class. Subsequently, Figure 7 presents the Receiver Operating Characteristic (ROC) of the proposed CNN model across all runs. The red line depicts the mean ROC curve, and the blue area indicates the performance variability across runs. The AUC is 0.997, indicating a high classification performance. Additionally, Table 4 provides detailed metrics for each grade classification, including True Positives (TP), True Negatives (TN), False Positives (FP), False Negatives (FN), Accuracy (Acc), Specificity (Sp), Sensitivity (Se), and Precision (Pr).

### 3.3. Effect of Augmentations on the Performance of the Proposed CNN

**Figure 8:**
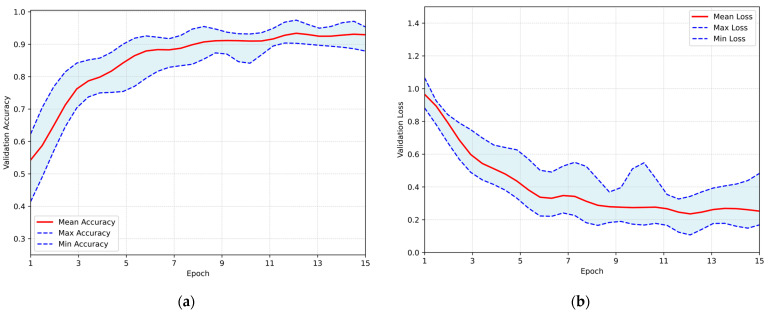
Overall performance of the proposed CNN, trained on the augmented dataset, evaluated using the Repeated Hold-Out validation strategy: (**a**) validation accuracy per training epoch; (**b**) validation loss per training epoch.

Mild augmentations were applied to the training sets to enhance the validation and generalization capability of the proposed architecture. This approach aimed to assess the proposed model’s performance under the influence of distorted representations of the dataset. The training results obtained by employing the same Repeated Hold-Out Validation strategy, are presented in Figure 8. The plot illustrates the validation accuracy and loss over the epochs, with the red line representing the mean performance and the shaded blue area indicating the performance range in both subfigures. The system demonstrated a peak average accuracy of 93.34% on the test set at the 12th epoch, with a standard deviation of ±2.07% and a corresponding 95% CI of 92.03–94.66%. At the same time, the model maintained a low loss rate, with the minimum average loss recorded at 0.236. This performance highlights the model’s robustness and its ability to generalize effectively to unseen data, even under augmented conditions.

**Figure 9:**
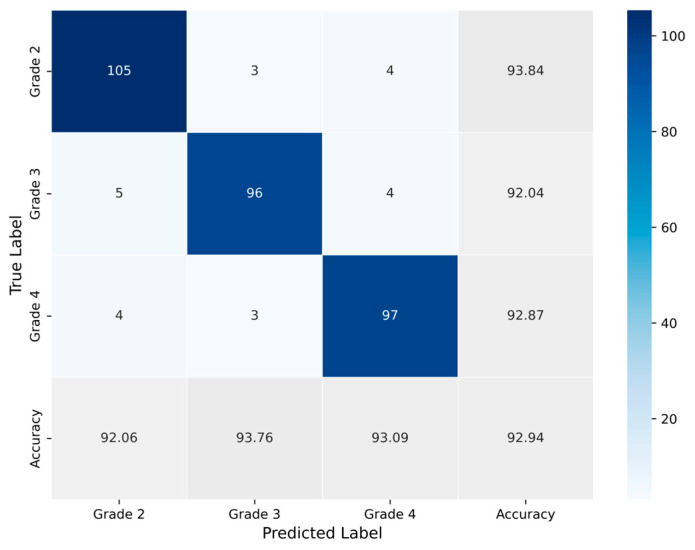
Average confusion matrix obtained across all runs of the Proposed CNN model trained on the augmented dataset, illustrating the classification performance for the three astrocytoma grades.

**Figure 10:**
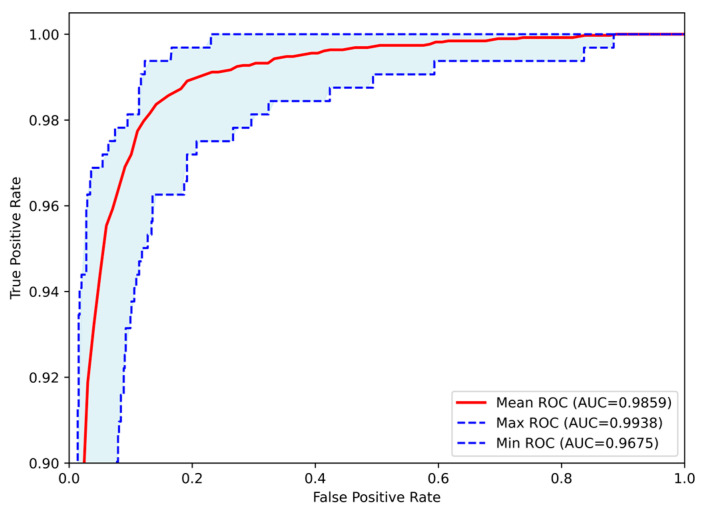
Receiver Operating Characteristic (ROC) curves of the Proposed CNN model for astrocytoma grade classification, trained on the augmented dataset and aggregated across all runs. The mean ROC (AUC = 0.9859) reflects the model’s overall discriminative performance, while the maximum (AUC = 0.9938) and minimum (AUC = 0.9675) curves indicate the range of variability across runs.

Subsequently, evaluation metrics were applied to assess the system’s overall performance. Figure 9 illustrates the confusion matrix, which displays the number of samples correctly and incorrectly classified per class after the augmented training. Additionally, Figure 10 presents the ROC-AUC curves derived from all runs after training with augmented data. The red line represents the mean ROC curve, while the shaded blue area indicates the performance range across the runs. The mean AUC is 0.986, maintaining a high level of classification performance, with minimal deviation from perfect classification, despite the altered dataset representations. Lastly, Table 5 summarizes the performance metrics derived from the confusion matrix for each class. Specifically, the table reports True Positives (TP), True Negatives (TN), False Positives (FP), False Negatives (FN), Accuracy (Acc), Specificity (Sp), Sensitivity (Se), and Precision (Pr), providing a detailed evaluation of the classification outcomes for each grade.

### 3.4. Comparison with State-of-the-Art Networks

To assess the effectiveness and highlight the advantages of the proposed method, a comparative analysis was conducted using four widely adopted pre-trained CNNs. These networks, including VGG-16 [35], AlexNet [36], ResNet-50 [37], and InceptionV3 [38], are considered state-of-the-art in medical imaging and deep learning applications, due to their consistently high performance. Each of these architectures follows a distinct design strategy, differing in depth, convolutional block arrangement, and feature extraction mechanisms, which result in varying approaches to processing and classifying images.

The input phase incorporated the identical pre-processing stages, and the evaluation employed the Repeated Hold-Out Validation technique to ensure a fair comparison between the proposed CNN and the benchmark models. Transfer Learning was implemented by initializing the networks with ImageNet pre-trained weights. Two experiments were conducted, the first with all base weights frozen and only the newly added fully connected layers trained, allowing adaptation to the characteristics of the target dataset. An additional full fine-tuning experiment was conducted, in which all layers were trainable, that led to lower performance. Therefore, the reported results correspond to the optimal performance achieved through the feature extraction strategy.

**Figure 11:**
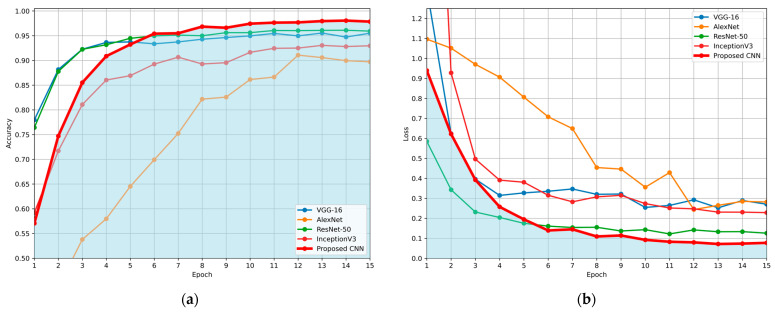
Comparison of the average evaluation metrics across training epochs for the proposed CNN and benchmark models (VGG-16, AlexNet, ResNet-50, and InceptionV3), using the Repeated Hold-Out validation technique: (**a**) average validation accuracy per epoch; (**b**) average validation loss per epoch. Blue areas in both (**a**) and (**b**) were created to highlight the performance of the proposed CNN, compared to the other networks.

**Figure 12:**
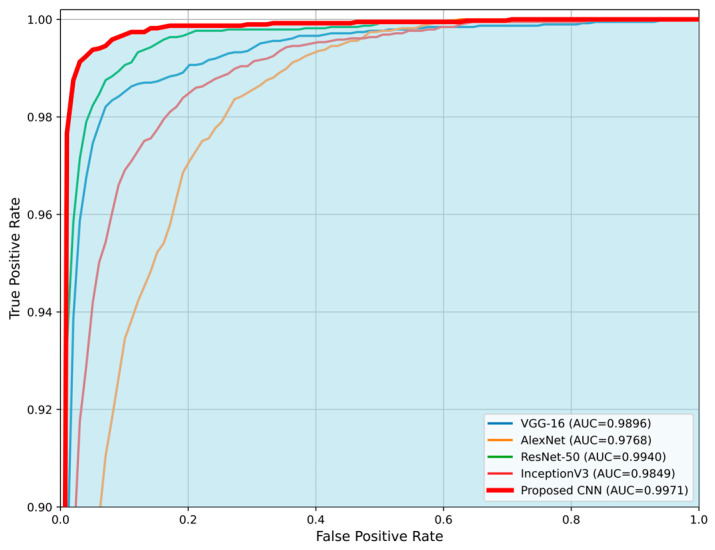
Comparison of the average Receiver Operating Characteristic (ROC) curves for the proposed CNN model and state-of-the-art architectures (VGG-16, AlexNet, ResNet-50, and InceptionV3). The proposed CNN demonstrates the highest Area Under the Curve (AUC = 0.9971), indicating superior classification performance. The blue area highlights the performance of the proposed CNN compared to the other networks.

The comparison is made in terms of average accuracy, loss, and AUC as shown in Table 6. Additionally, Figure 11 and Figure 12 present a visual comparison of the training metrics. Figure 11 illustrates the average accuracy and the average loss of each network, respectively, across the training epochs. After the 6th epoch, the proposed CNN demonstrates superior overall performance, surpassing ResNet-50, which initially exhibited the best performance among the pre-trained models. Figure 12 presents a comparison of the average ROC-AUC across all runs between the proposed CNN and the state-of-the-art networks, highlighting the proposed CNN’s highest AUC value derived from the ROC curve.

**Figure 13:**
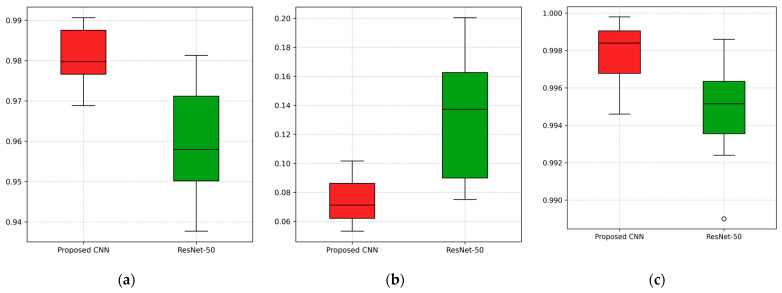
Visualization of the comparison between the proposed CNN and the second-best performing model, ResNet-50, across 12 independent runs for (**a**) validation accuracy, (**b**) validation loss, and (**c**) AUC value.

To further validate the efficacy of the proposed CNN over the ResNet-50, which achieved the second-highest performance, a statistical comparison was conducted. The Wilcoxon Signed Rank Test is a non-parametric test that is appropriate for evaluating the performance of deep learning models, where the assumption of normality is not satisfied [39,40]. The performances of the two networks were evaluated pairwise over the 12 independent runs, using the results obtained at the 14th epoch (over 15), which corresponds to the peak performance for both networks. The comparison was based on validation accuracy, validation loss and AUC value. The resulting *p*-value for accuracy, loss and AUC were 0.001, 0.001, and 0.005, respectively, indicating statistically significant differences (*p* < 0.01) in favor of the proposed model. Figure 13 presents boxplots to visualize the comparison between the proposed CNN and ResNet-50 in terms of validation accuracy, validation loss, and AUC.

### 3.5. Explainable AI Insights

The final stage of this study focuses on the application of Explainable AI techniques to interpret the decision-making process of the proposed CNN. This approach ensures that the model’s classification is based on relevant tumor grade characteristics and not influenced by confounding parameters, such as differences between MRI sequences or potential image artifacts. By providing transparency in the model’s decisions, the system transitions from a ‘black box’ to a valuable tool that encourage clinicians to incorporate it in decision-making process.

**Figure 14:**
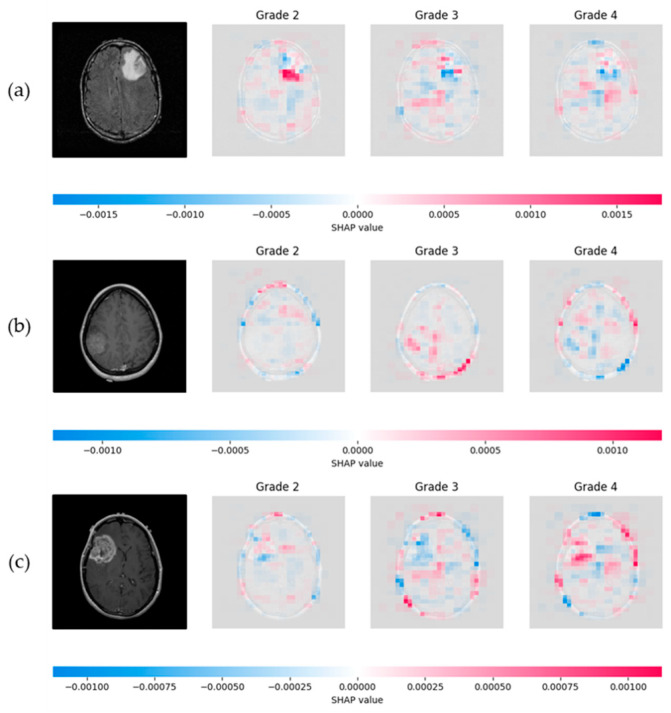
SHAP visualization illustrating the influence of slice pixels through the proposed network, providing transparency on astrocytoma grading. (**a**) Visualization of a Grade 2 astrocytoma sample; (**b**) Grade 3 astrocytoma; (**c**) Grade 4 astrocytoma. For each case, SHAP values are presented for all three possible class predictions. Red regions indicate positive contributions toward the predicted class, while blue regions indicate negative contributions associated with features of other grades. Color intensity reflects the magnitude of each pixel’s influence.

The SHAP model was applied to interpret the network’s decisions, utilizing the Shapley values to provide both local and global explanations, thereby assessing the overall system’s performance. The number of evaluations was fixed to 50,000, ensuring an accurate and comprehensive evaluation of the model’s decision-making process. Figure 14 illustrates the application of SHAP, where the proposed CNN classified one representative test sample from each astrocytoma grade (G2–G4). For each input slice, SHAP visualizations were generated for all three possible tumor grades, enabling a detailed understanding of the network’s behavior. In these visualizations, red areas represent 2D cross-sectional regions with a positive contribution toward the predicted class, while blue areas include features that correspond to other classes’ slice characteristics, indicating a negative correlation. The intensity of the red and blue colors reflects the strength of these contributions.

**Figure 15:**
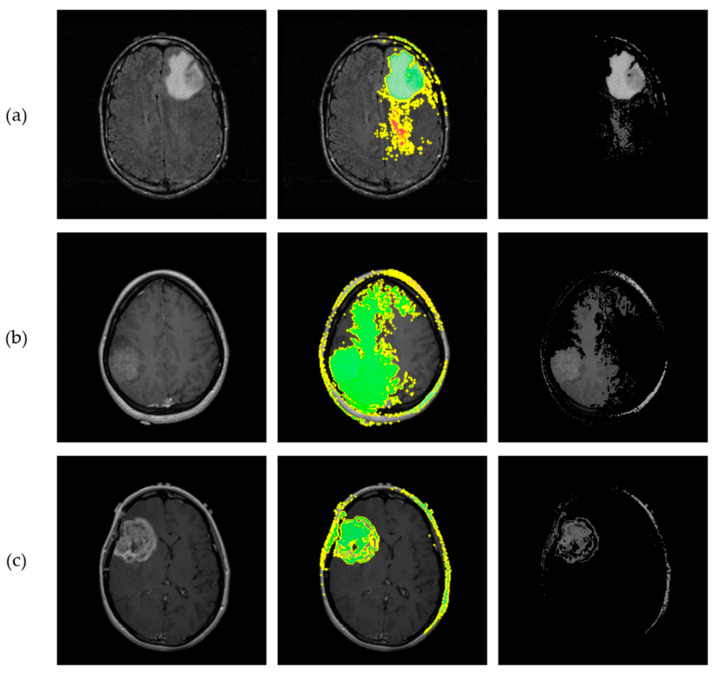
Results from implementation of the LIME algorithm, illustrating the local contributions identified by the proposed CNN and offering interpretability for the astrocytoma grading process. Each row corresponds to a different tumor grade. (**a**) Visualization of a Grade 2 astrocytoma sample; (**b**) Grade 3 astrocytoma; (**c**) Grade 4 astrocytoma. From left to right: original MRI slice, LIME explanation highlighting the top 3 contributing regions, and masked original slice where only the top regions are retained. Green and Red colors indicate positive and negative contribution of the corresponding “superpixels” for class prediction, respectively.

LIME was subsequently employed as a heuristic Explainable AI tool to interpret the proposed CNN’s predictions. To generate local explanations, LIME segmented each 2D slice into superpixels and produced 20,000 perturbed samples per instance. The analysis focused on the three most influential regions contributing to each prediction. LIME then visualizes these regions by applying a green or red color overlay, indicating whether the region contributes positively or negatively to the predicted class, respectively. Additionally, a masked version of the original slice was produced, where only the most influential regions were retained, offering a clear view of the areas contributed to model’s decisions. Figure 15 illustrates the application of LIME algorithm on the same representative astrocytoma samples used in the SHAP analysis.

**Figure 16:**
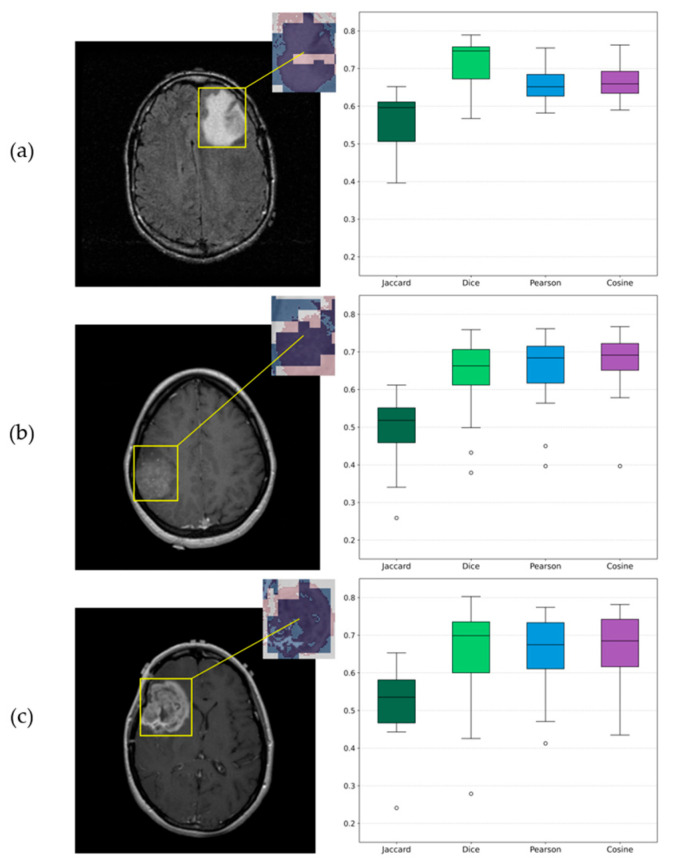
Visualizations of similarity analysis results between SHAP and LIME algorithms for (**a**) Grade 2 astrocytoma sample; (**b**) Grade 3 astrocytoma; (**c**) Grade 4 astrocytoma. From left to right, the ROI is highlighted on the corresponding original slice, a zoomed-in ROI view of SHAP and LIME mean maps overlaid in red and blue, respectively, and the corresponding boxplots provide a visualization of distributions.

Beside the visualizations that provide a qualitative perspective for assessing each algorithm’s results, a quantitative analysis was conducted to examine their agreement across the 12 independent runs. The outcomes of this evaluation are summarized in Table 7, which presents the average similarity metrics along with the corresponding sample-based standard deviations between SHAP and LIME within the tumor regions. Additionally, a representative visualization of the mean overlay for the three examples is presented in Figure 16. From left to right, each row shows the original slice with the corresponding ROI, a zoomed-in view of the same ROI with SHAP and LIME mean maps overlaid in the red and blue channels, respectively, and boxplots derived from the similarity metrics, providing a visual representation of their distribution across experiments.

## 4. Discussion

Magnetic Resonance Imaging (MRI) is the primary non-invasive modality used for the initial assessment and grading of glioma. MRI acquisitions from multiple sequences, including T1-, T2-weighted, Fluid-Attenuated Inversion Recovery (FLAIR), and contrast-enhanced (CE) images, provide comprehensive anatomical and tissue characterization information that aids in tumor detection, localization, and assessment [41].

The increasing availability of imaging data has enabled the application of Machine Learning (ML) techniques to automate the classification of gliomas, thereby enhancing diagnosis support and decision-making processes. A literature review of studies from 2010 to 2019 highlighted an upward trend in research based on glioma grade prediction, utilizing deep learning approaches. Notably, in the later years of this period, deep learning methods and particularly Convolutional Neural Networks (CNNs) demonstrated better performance compared to traditional ML algorithms [42,43].

In this context, the proposed CNN demonstrated excellent classification performance, achieving a validation accuracy of 98.05%, accompanied by a small standard deviation across runs, low validation loss and a near-perfect ROC-AUC score of 0.9971. These results indicate a highly reliable model capable of accurately distinguishing between astrocytoma grades.

Data augmentation techniques were applied to the training set by replacing it with an altered version for validation purposes. The training results demonstrated that the CNN maintained high validation accuracy on the original test set, despite the applied alterations. This outcome shows the robustness of the network architecture, while this methodology has a high impact on astrocytoma grade classification.

A comparative evaluation was performed between the proposed CNN and several state-of-the-art architectures utilizing transfer learning. All models were trained and evaluated under identical conditions to ensure a fair comparison. The proposed approach outperformed the pre-trained models, underscoring the advantage of a fully optimized architecture specifically designed for the field of brain tumor imaging. Notably, the proposed model demonstrated steady and repeatable high performance, exhibiting the lowest standard deviation among the pre-trained models. This finding highlights those custom architectures, designed with domain-specific data in mind, can surpass generic models trained on large-scale natural image datasets. Furthermore, the application of Wilcoxon Signed-Rank Test revealed a statistically significant difference (*p* < 0.01) between the proposed CNN and ResNet-50, the second-best performing model, further validating the effectiveness and reliability of the proposed approach.

Explainable AI (X-AI) methods were also integrated into the study to enhance transparency and interpretability. Both SHAP and LIME algorithms provided visual explanations, highlighting common regions of high importance across samples. The subsequent similarity analysis further quantified this agreement, showing that the models were largely consistent, with all mean Jaccard values above 0.5 and Dice, Pearson and Cosine metrics above 0.6, despite the presence of two outlier runs. This outcome is reasonable given the different mechanisms of the two algorithms. Overall, this agreement confirms that the model’s predictions were based on meaningful tumor-related regions and demonstrates its ability to accurately identify and intergrade these critical areas into decision-making process.

Despite the promising results, a significant limitation of this study is the absence of validation on an independent external dataset. While internal validation showed excellent performance, it is essential to confirm the model’s ability to generalize to unseen astrocytoma cases. This constraint arises, in part, from the limited size of the available dataset, which prevented the allocation of an independent subset without substantial loss of training information. Another limitation is the challenge of training under a patient-wise split due to the small number of patients in the dataset. Additionally, the CNN was trained to classify the whole tumor region and did not explicitly distinguish between tumor subregions such as peritumoral edema, cystic areas, or regions of angiogenesis, as the datasets did not provide annotated subregions and expert guidance would be required for such segmentations. A promising future direction for research would involve the collection of multimodal data from local institutions, which could enhance clinical applicability and allow incorporation of subregion information.

Finally, a comprehensive comparison with prior studies is presented in Table 8 Regarding the performance of the proposed model, the results demonstrate competitive and effective behavior within the broader field of glioma classification. Specifically for astrocytoma grading, the proposed approach achieved the highest metrics in distinguishing malignant astrocytic tumor grades. Additionally, this study differs significantly by minimizing pre-processing requirements and focusing on model interpretability, an area that was not included or minimally addressed in previous works. System’s transparency provides an extra value to the diagnostic process, enhancing the trust of clinicians.

## 5. Conclusions

In summary, this study presents the development of a fully optimized CNN framework for astrocytoma grading based on 2D cross-sectional MRI scans. The results demonstrated high predictive performance, strong repeatability, and interpretability. By combining architecture and hyperparameter optimization through the Hyperband method, the model is capable of analyzing slices with minimal or no pre-processing, providing reliable diagnostic support. Additionally, emphasis was placed on robust system validation by applying augmentations exclusively to training sets, in order to evaluate the performance and generalization ability under altered input conditions. The application of XAI techniques enhances the transparency and trust, preventing the system from functioning as a black box.

## Figures and Tables

**Table 1:** Search space of architectural hyperparameters and optimal values obtained using the Hyperband algorithm.

Architectural Hyperparameters Optimization			
Layer	Hyperparameter	Search Range	Optimal Value
Layer 1	Number of filters	[32, 64, 96, 128, 160, 192, 224, 256]	128
	Filter Size	[(3, 3), (4, 4), (5, 5), (6, 6)]	(6, 6)
	Max Pooling Layer	[True, False]	False
Layer 2	Number of filters	[32, 64, 96, 128, 160, 192, 224, 256]	32
	Filter Size	[(3, 3), (4, 4), (5, 5), (6, 6)]	(3, 3)
	Max Pooling Layer	[True, False]	True
Layer 3	Number of filters	[32, 64, 96, 128, 160, 192, 224, 256]	96
	Filter Size	[(2, 2), (3, 3), (4, 4)]	(4, 4)
	Max Pooling Layer	[True, False]	False
Layer 4	Number of filters	[32, 64, 96, 128, 160, 192, 224, 256]	96
	Filter Size	[(2, 2), (3, 3), (4, 4)]	(3, 3)
	Max Pooling Layer	[True, False]	True
Layer 5	Number of filters	[32, 64, 96, 128, 160, 192, 224, 256]	224
	Filter Size	[(2, 2), (3, 3), (4, 4)]	(3, 3)
	Max Pooling Layer	[True, False]	False
Layer 6	Number of filters	[32, 64, 96, 128, 160, 192, 224, 256]	32
	Filter Size	[(2, 2), (3, 3), (4, 4)]	(4, 4)
	Max Pooling Layer	[True, False]	True
Layer 1–6	Strides	[(1, 1), (2, 2)]	(1, 1)
	Padding	[Same, Valid]	Valid
FC Layer	Units	[64, 96, 128, 160, 192, 224, 256, 288, 320, 352, 384, 416, 448, 480, 512]	480

**Table 2:** Search space of regularization hyperparameters and optimal values obtained using the Hyperband algorithm.

Regularization Hyperparameters Optimization		
Hyperparameter	Search Range	Optimal Value
Dropout Rate	[0.2, 0.3, 0.4, 0.5]	0.2

**Table 3:** Search space of training hyperparameters and optimal values obtained using the Hyperband algorithm.

Training Hyperparameters Optimization		
Hyperparameter	Search Range	Optimal Value
Learning Rate	[0.0001, 0.0005, 0.001]	0.0001
Batch Size	[16, 32, 64]	32

**Table 4:** Classification performance metrics of the Proposed CNN model across all runs, derived from the confusion matrix. The table reports True Positives (TP), True Negatives (TN), False Positives (FP), False Negatives (FN), Accuracy (Acc), Specificity (Sp), Sensitivity (Se), and Precision (Pr) for each astrocytoma grade.

**Classes**	**TP**	**TN**	**FP**	**FN**	**Acc (%)**	**Sp (%)**	**Se (%)**	**Pr (%)**
Grade 2	110	205	3	2	98.44	98.56	98.21	97.35
Grade 3	98	217	2	3	98.44	99.09	97.03	98.00
Grade 4	106	212	1	1	99.38	99.53	99.06	99.06

**Table 5:** Classification performance metrics of the Proposed CNN model across all runs, trained on augmented dataset and derived from the confusion matrix. The table reports True Positives (TP), True Negatives (TN), False Positives (FP), False Negatives (FN), Accuracy (Acc), Specificity (Sp), Sensitivity (Se), and Precision (Pr) for each astrocytoma grade.

Classes	TP	TN	FP	FN	Acc (%)	Sp (%)	Se (%)	Pr (%)
Grade 2	105	200	9	7	95.02	95.69	93.75	92.10
Grade 3	96	210	6	9	95.33	97.22	91.43	94.12
Grade 4	97	209	8	7	95.33	96.31	93.27	92.38

**Table 6:** Performance Comparison with State-of-the-art Networks. Accuracy is reported as mean ± standard deviation, with the corresponding 95% CI shown in brackets. Loss and AUC are reported as mean values.

Network	Accuracy (%)	Loss	AUC
VGG-16	95.53 ± 1.75 [91.61–94.48]	0.2523	0.9896
AlexNet	91.07 ± 2.71 [89.35–92.79]	0.2436	0.9768
ResNet-50	96.11 ± 1.38 [95.23–96.99]	0.1217	0.9940
InceptionV3	93.04 ± 2.26 [91.61–94.48]	0.2283	0.9849
Proposed CNN	98.05 ± 0.72 [97.60–98.51]	0.0713	0.9971

**Table 7:** Similarity metrics between SHAP and LIME maps within the ROIs across runs of the Proposed CNN. Values reported as mean ± standard deviation, with the corresponding 95% C.I. shown in brackets.

Sample	Jaccard	Dice	Pearson	Cosine
Grade 2	0.56 ± 0.08 [0.51–0.61]	0.72 ± 0.07 [0.68–0.76]	0.66 ± 0.05 [0.63–0.69]	0.67 ± 0.05 [0.64–0.70]
Grade 3	0.50 ± 0.10 [0.44–0.56]	0.63 ± 0.11 [0.56–0.70]	0.64 ± 0.11 [0.57–0.71]	0.65 ± 0.12 [0.57–0.73]
Grade 4	0.51 ± 0.10 [0.45–0.57]	0.64 ± 0.14 [0.55–0.73]	0.65 ± 0.10 [0.59–0.71]	0.66 ± 0.11 [0.59–0.73]

**Table 8:** Comparative analysis of classification performance (accuracy and AUC) across the proposed model and previous studies.

Author	Year	Classification Type	Accuracy AUC	Data Collection
[7]	2023	LG-Gliomas HG-Gliomas	96.40%, 98.52% -	BraTS-2017, Department of Radiology, BVHB, Pakistan
[8]	2023	LG-Gliomas HG-Gliomas	99.85% 0.9992	BraTS-2020
[9]	2022	G-2 Gliomas G-3 Gliomas G-4 Gliomas	97.14% -	REMBRANDT
[10]	2024	G-2 Gliomas G-3 Gliomas G-4 Gliomas	98.56% 0.9993	RIDER REMBRANDT TCGA-LGG
[11]	2021	LG-Astrocytomas Anaplastic Astrocytomas	72.90% 0.825	Department of Neurosurgery and the Cancer Centre of West China Hospital
[12]	2021	G-1 Astrocytomas G-2 Astrocytomas G-3 Astrocytomas G-4 Astrocytomas	96.56% -	Department of Radiology, BVHB, Pakistan
Proposed Method	2025	G-2 Astrocytomas G-3 Astrocytomas G-4 Astrocytomas	98.05% 0.9971	REMBRANDT TCGA-LGG

## Data Availability

The original imaging data utilized and processed in this study are openly available in The Cancer Imaging Archive (TCIA). The REMBRANDT collection is available at [14], and the TCGA-LGG collection is available at [15].

## Abbreviations

The following abbreviations are used in this manuscript:

AAI	Artificial Intelligence Act
AUC	Area Under the Curve
CE	Contrast Enhanced
CI	Confidence Interval
CNS	Central Nervous System
CNN	Convolutional Neural Network
FLAIR	Fluid-Attenuated Inversion Recovery
HGG	High Grade Glioma
LGG	Low Grade Glioma
LIME	Local Interpretable Model-Agnostic Explanations
MRI	Magnetic Resonance Imaging
REMBRANDT	Repository of Molecular Brain Neoplasia Data
RGB	Red, Green, Blue
ROI	Region of Interest
ROC	Receiver Operating Characteristic
SHAP	SHapley Additive exPlanations
TCGA-LGG	The Cancer Genome Atlas Low Grade Glioma
TCIA	The Cancer Imaging Archive
WHO	World Health Organization
XAI	Explainable Artificial Intelligence
